# Endoscopic gallbladder stenting for acute cholecystitis: a retrospective study of 46 elderly patients aged 65 years or older

**DOI:** 10.1186/1471-230X-13-65

**Published:** 2013-04-12

**Authors:** Satoshi Maekawa, Ryosuke Nomura, Takayuki Murase, Yasuyoshi Ann, Masayuki Oeholm, Masaru Harada

**Affiliations:** 1 Department of Gastroenterological Medicine, Japan Labour Health and Welfare Organization Niigata Rosai Hospital, 1-7-12 Touncho, Joetsu, Niigata, 942-8502, Japan; 2 Third Department of Internal Medicine, University of Occupational and Environmental Health, 1-1 Iseigaoka, Yahata-nishi-ku, Kitakyushu, Fukuoka, 807-8555, Japan

**Keywords:** Endoscopic gallbladder stenting, Cholecystitis, Percutaneous transhepatic gallbladder drainage, percutaneous transhepatic gallbladder aspiration, Endoscopic transpapillary naso-gallbladder drainage, Elderly patients

## Abstract

**Background:**

Endoscopic transpapillary pernasal gallbladder drainage and endoscopic gallbladder stenting (EGS) have recently been reported to be useful in patients with acute cholecystitis for whom a percutaneous approach is contraindicated. The aim of this study was to evaluate the efficacy of permanent EGS for management of acute cholecystitis in elderly patients who were poor surgical candidates.

**Methods:**

We retrospectively studied 46 elderly patients aged 65 years or older with acute cholecystitis who were treated at Japan Labour Health and Welfare Organization Niigata Rosai Hospital. In 40 patients, acute cholecystitis was diagnosed by transabdominal ultrasonography and computed tomography, while 6 patients were transferred from other hospitals after primary management of acute cholecystitis. All patients underwent EGS, with a 7Fr double pig-tail stent being inserted into the gallbladder. If EGS failed, percutaneous transhepatic gallbladder drainage or percutaneous transhepatic gallbladder aspiration was subsequently performed. The main outcome measure of this study was the efficacy of EGS.

**Results:**

Permanent EGS was successful in 31 patients (77.5%) with acute cholecystitis, without any immediate postprocedural complications such as pancreatitis, bleeding, perforation, or cholangitis. The most common comorbidities of these patients were cerebral infarction (n=14) and dementia (n=13). In 30 of these 31 patients (96.7%), there was no recurrence of cholecystitis and 29 patients (93.5%) remained asymptomatic until death or the end of the study period (after 1 month to 5 years).

**Conclusions:**

EGS can be effective for elderly patients with acute cholecystitis who are poor surgical candidates and can provide a solution for several years.

## Background

Acute calculous cholecystitis appears to arise from obstruction of the cystic duct or the junction between the gallbladder and cystic duct by a stone or edema resulting from local mucosal erosion and inflammation caused by a stone [1]. Early cholecystectomy is the standard therapy for acute cholecystitis [1,2]. Although cholecystectomy is generally safe, its mortality rate increases markedly in high-risk patients with comorbidities [3,4]. In critically ill elderly patients, the mortality rate of emergency cholecystectomy can be as high as 30% [3,4]. Therefore, as a temporary measure, high-risk patients are treated by decompression of the gallbladder using percutaneous transhepatic gallbladder drainage (PTGBD) or percutaneous transhepatic gallbladder aspiration (PTGBA) [5-8]. In addition, several authors have reported that endoscopic transpapillary naso-gallbladder drainage (ETGBD) is a safe and effective procedure for acute cholecystitis [9-12]. Endoscopic gallbladder stenting (EGS) may not only be effective for acute cholecystitis, but could also be a viable strategy for long-term management of symptomatic cholelithiasis in patients who are poor surgical candidates [13-24]. However, very few studies have provided detailed long-term follow up analysis of EGS.

Here, we report our experience with various EGS techniques for the management of acute cholecystitis and evaluate the efficacy of permanent stenting as an option in elderly patients who are poor surgical candidates.

## Methods

The study population consisted of 46 patients with acute cholecystitis who were 65 years of age or older. They were admitted to Japan Labour Health and Welfare Organization Niigata Rosai Hospital between 2007 and 2012. In 40 patients, acute cholecystitis was diagnosed by transabdominal ultrasonography (US) and computed tomography (CT) at our hospital, while 6 patients were transferred from other hospitals after primary management of acute cholecystitis. After endoscopic retrograde cholangiography (ERC), EGS was attempted in all 46 patients.

ERC was performed with a video duodenoscope (JF-260V; Olympus Medical Systems, Tokyo, Japan). Cannulation of the bile duct and cystic duct was done with an over-the-wire endoscopic retrograde cholangiopancreatography (ERCP) cannula (Tandem XL; Boston Scientific, Tokyo, Japan) and an 0.018, 0.025, or 0.035 inch guidewire (Radifocus; Terumo Tokyo, Japan or Chiarida; Century Medical Ink, Tokyo, Japan). The guidewire was advanced retrogradely through the cannula and coiled within the gallbladder. Over this wire, a 7Fr double pig-tail stent (10 or 15 cm, Olympus) was advanced into the gallbladder (Additional file 1).

Clinical success with EGS was defined as complete resolution of symptoms, improvement of laboratory data (white blood cell count and C-reactive protein), and improvement of US findings. Follow-up of permanent EGS patients was performed until death or the end of the study period, with review of symptoms and laboratory data every 3 months plus US every 6 months.

Written informed consent was obtained from the patients (or from a family member if the patient had cerebral infarction or dementia) and the ethics committee of Japan Labour Health and Welfare Organization Niigata Rosai Hospital approved this study. We obtained consent for publication of all material in this article, including the individual information contained in the tables.

## Results

The characteristics of the 46 subjects are depicted in Table  1. Their average age was 79.70±7.96 years (mean ± standard deviation [SD]). Twenty-four patients also had choledocholithiasis, and we performed endoscopic sphincterotomy (EST) in 34 patients, including 10 without choledocholithiasis to obtain adequate post-EGS flow in the common bile duct. Emergency EGS was successful in 31 out of 40 patients (77.5%) with acute cholecystitis. We also performed elective EGS for the purpose of permanent placement in 6 patients. We inserted a 10 cm double pig-tail stent in 7 patients and a 15 cm double pigtail stent in 30 patients. There were no immediate postprocedural complications, such as pancreatitis, bleeding, perforation, or cholangitis.

**Table 1:** Characteristics of the patients receiving EGS

**No. of patients**	**46**
Age (years, mean ± SD)	79.70±7.96
Gender (M/F)	25/21
Cause of cholecystitis	
Stone	40
Sludge	6
Choledocholithiasis	24

EGS = endoscopic gallbladder stenting.

**Figure 1:**
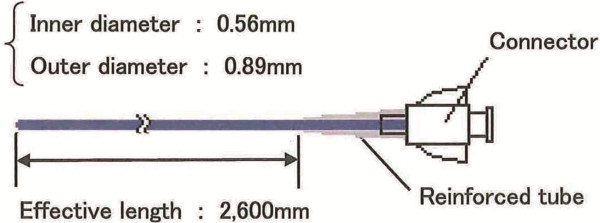
The structure of the microcatheter used for endoscopic gallbladder stenting.

**Figure 2:**
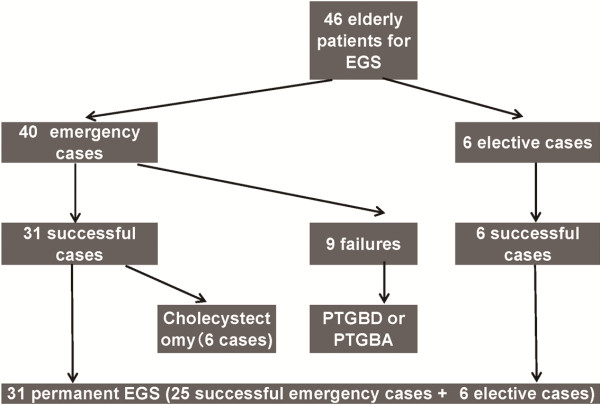
**Outcome of 46 elderly patients with acute cholecystitis received EGS.** EGS, endoscopic gallbladder stenting; PTGBD, percutaneous transhepatic gallbladder drainage; PTGBA, percutaneous transhepatic gallbladder aspiration

We performed emergency EGS in 40 patients who presented with acute severe cholecystitis and were poor candidates for cholecystectomy. In 31 patients (77.5%), emergency EGS was successful and the procedural time was 27.6 ± 15.1 min (mean ± SD). Clinical success was achieved within 3 days in all 31 patients. Their symptoms (abdominal pain, fever, and vomiting) resolved and they started to eat from the day after emergency EGS, and all 31 showed normalization of WBC and a decrease of C-reactive protein after 3 days. All were discharged from hospital within 1 week. We administered antibiotics to all 31patients for 3 days after emergency EGS, but they did not need ICU support, intubation, or inotropes. Among the 31 successful patients, we used a microcatheter for EGS in 4 difficult cases. The reasons for difficulty were severe stricture and kinking of the cystic duct and we accomplished EGS in these cases by using an 0.018 inch guidewire and a microcatheter (Additional file 2). The microcatheter had an inner diameter of 0.59 mm, outer diameter of 0.89 mm, and an effective length of 2,600 mm (Figure 1). Of the 31 patients, 6 patients underwent cholecystectomy within 2 months of EGS, while 25 patients were followed up without surgery due to their poor general condition (Figure 2).

Among the 40 patients in whom emergency EGS was attempted, it failed in 9 patients because the cystic duct branched caudally from the common bile duct (n=3), because there was severe kinking of the cystic duct (n=3), because the cystic duct was completely blocked by stones (n=2), or because of cystic duct adhesions (n=1). Among the 9 patients in whom EGS failed, we performed PTGBD in 6 patients and repeated PTGBA in 3 patients. Of the 6 patients receiving PTGBD, 5 underwent cholecystectomy within 2 months, while 1 patient died of aspiration pneumonia after 1 month. The 3 patients receiving repeated PTGBA were discharged from hospital after 2 or 3 weeks.

**Figure 3:**
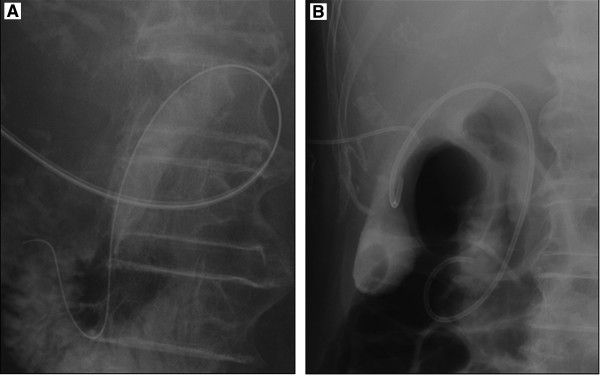
A guidewire has been passed antegradely into the duodenum via the PTGBD route (A), and EGS was performed by using the guidewire retrogradely (B).

We also performed elective EGS for the purpose of permanent placement in 6 patients (5 after PTGBD and 1 after conservative treatment) who were transferred from other hospitals within 1 week after the onset of acute cholecystitis, and the procedurewas successful in all 6 patients. In 5 patients, EGS was done by passing the guidewire antegradely into the duodenum via the PTGBD route and then the guidewire was used retrogradely (Figure 3). In 1 patient, EGS was done via peroral cholangioscopy (POCS) with a GIF-XP260 (Olympus) because the cystic duct branched caudally from the common bile duct and this was considered to be a very difficult case for EGS. The procedure was started by inserting the GIF-XP260 into the common bile duct after endoscopic sphincterotomy. We endoscopically explored the cystic duct and then easily inserted the guidewire and the cannula into the duct. Then the guidewire was advanced retrogradely through the cannula and coiled within the gallbladder. Afterwards, we exchanged the GIF-XP260 for the GIF-Q260, and a double pigtail stent was advanced into the gallbladder over the wire (Additional file 3).

Permanent EGS was useful for prevention of cholecystitis in 31 elderly patients who were poor surgical candidates. Their clinical characteristics and outcomes are listed in Table  2. The average age of the patients was 82.19 ± 7.15 years (mean ± SD). Sequelae of cerebral infarction were present in 14 patients, dementia in 13 patients, severe heart disease in 1 patient, cerebral hemorrhage in 1 patient, malignant mesothelioma in 1 patient, and polymyalgia rheumatica in 1 patient. The follow-up period ranged from 1 month to 5 years. In 30 of 31patients (96.7%), there was no recurrence of cholecystitis and 29 patients (93.5%) remained asymptomatic for 1 month to 5 years (until death or the end of the study period). Sixteen patients (51.6%) died from 1 month to 5 years after the procedure due to aspiration pneumonia, heart failure, cerebral infarction, gastric cancer, or malignant mesothelioma. Two patients developed late complications. Patient no. 3 developed vomiting at 2 months and 8 months after EGS. We diagnosed her as acute cholecystitis from the US and CT findings (occlusion at 2 months and migration at 8 months). After the stent was exchanged with ERC, the symptoms soon resolved. Following the second exchange, there have been no further complications. Patient no. 5 underwent removal of the gallbladder stent at 3.5 years after EGS because he developed a liver abscess (without cholecystitis). He was treated with antibiotics and the abscess resolved after 2 months. We did not reinsert a gallbladder stent because the patient refused it. Although scheduled stent exchange was not done, the other 29 patients (93.5%) did not have any late complications.

**Table 2:** Outcome of stenting

**Patient no.**	**Age (yrs)**	**Gender**	**Other diseases**	**Cause of cholecystitis**	**Stent exchange or removal**	**Outcome, time from initial stenting**
1	65	M	Cerebral infarction (sequelae)	Gallstone	No	Died of aspiration pneumonia (1 month)
2	79	M	Cerebral infarction (sequelae)	Gallstone	No	Alive (60 months)
3	79	F	Cerebral infarction (sequelae)	Sludge	Yes	Stent occlusion (2 months) and stent migration (8 months): alive after 2 exchanges (59 months)
4	88	F	Cerebral infarction (sequelae)	Gallstone	No	Died of aspiration pneumonia (56 months)
5	77	M	Severe heart disease	Gallstone	Yes	Liver abscess (44 months): alive after drainage (57 months)
6	91	F	Cerebral infarction (sequelae)	Gallstone	No	Died of gastric cancer, 24 months
7	85	F	Dementia	Gallstone	No	Alive (56 months)
8	86	F	Dementia	Gallstone	No	Died of aspiration pneumonia (11 months)
9	93	M	Dementia	Gallstone	No	Died of aspiration pneumonia (51 months)
10	78	M	Dementia	Gallstone	No	Alive (52 months)
11	85	M	Cerebral infarction (sequelae)	Sludge	No	Died of heart failure (22 months)
12	76	F	Cerebral infarction (sequelae)	Sludge	No	Alive (52 months)
13	83	M	Cerebral infarction (sequelae)	Sludge	No	Died of heart failure (2 months)
14	93	M	Dementia	Gallstone	No	Died of heart failure (26 months)
15	88	F	Cerebral infarction (sequelae)	Gallstone	No	Died of heart failure (35 months)
16	84	M	Dementia	Gallstone	No	Died of aspiration pneumonia (2 months)
17	74	M	Cerebral infarction (sequelae)	Gallstone	No	Alive (48 months)
18	86	M	Cerebral infarction (sequelae)	Gallstone	No	Alive (46 months)
19	73	M	Pleura Malignant Mesothelioma	Gallstone	No	Died of malignant mesothelioma (7 months)
20	75	M	Cerebral infarction (sequelae)	Gallstone	No	Died of aspiration pneumonia (16 months)
21	76	M	Cerebral hemorrhage (sequelae)	Gallstone	No	Alive (44 months)
22	94	F	Dementia	Gallstone	No	Alive (41 months)
23	79	F	Cerebral infarction (sequelae)	Gallstone	No	Died of aspiration pneumonia (1 month)
24	93	F	Dementia	Gallstone	No	Died of heart failure (26 months)
25	71	M	Dementia	Gallstone	No	Alive (17 months)
26	82	F	Polymyalgia rheumatica	Gallstone	No	Died of aspiration pneumonia (5 months)
27	82	M	Dementia	Gallstone	No	Died of cerebral infarction (29 months)
28	85	F	Dementia	Gallstone	No	Alive (48 months)
29	86	M	Dementia	Gallstone	No	Alive (43 months)
30	76	F	Dementia	Gallstone	No	Alive (26 months)
31	86	F	Cerebral infarction (sequelae)	Gallstone	No	Alive (11 months)

## Discussion

The definitive treatment for acute cholecystitis is cholecystectomy [1,2], but in critically ill elderly patients, the mortality rate of emergency cholecystectomy can reach 30% [3,4]. Several authors have described the usefulness of PTGBD or PTGBA for such poor surgical candidates [5-8]. However, we sometimes encounter patients who cannot tolerate the percutaneous transhepatic approach (e.g., because of anticoagulant/antiplatelet therapy, disseminated intravascular coagulation, gallbladder malposition, or severe contracture). Such patients are likely to develop intra-abdominal bleeding, biloma or even biliary peritonitis, if these procedures are performed. Recently, ETGBD has been reported as an alternative to PTGBD or PTGBA [9-12]. Although it is possible to wash the gallbladder via an ETGBD tube, there is a danger of the tube being pulled out by elderly patients and it cannot be left in the gallbladder over the long-term.

Otherwise EGS could be a viable strategy for long-term management of symptomatic cholelithiasis in patients who are poor surgical candidates. EGS has been shown to be an effective long-term option in cirrhosis patients with cholelithiasis as a bridge to liver transplantation [13-17]. Studies on EGS have demonstrated a favorable clinical outcome up to 3 years after stenting without any need for routine replacement of the stent [14,15]. In a prospective follow-up study, Lee et al. [18] also showed that at least 80% of 20 patients undergoing EGS maintain stent patency without requiring stent exchange for at least 2 years. In the present series, we performed permanent EGS in 31 elderly patients. As a result, 30 patients (96.7%) had no recurrence of cholecystitis and 29 patients (93.5%) remained asymptomatic for the duration of their survival. There were no late complications in 14 patients after follow up for more than 3 years, 9 patients after follow up for more than 4 years, and 1 patient after follow up for more than 5 years. Accordingly, this study has demonstrated the longest good clinical outcome for up to 5 years after EGS in comparison with earlier studies without routine stent replacement [10,12]. Stents are thought to protect against recurrent cholecystitis by occupying the lumen of the cystic duct, thereby preventing stone impaction [24]. It has also been postulated that after probable occlusion of the stent, adequate flow of bile from the gallbladder to the duodenum still occurs along the outer surface of the stent through capillary action, a phenomenon known as “wicking” [24]. Moreover, the stent may prevent bile flow from the common hepatic duct into the cystic duct. In our series, we found that the gallbladders of 12 patients became very small.

Only 2 patients developed late complications. One patient (Patient no. 3) had 2 episodes of recurrent cholecystitis at 2 months and 8 months after EGS (stent occlusion at 2 months and stent migration at 8 months). When occlusion occurred at 2 months, we thought that the probable cause was placing the stent without washing sludge out of the gallbladder with saline. After that, we had no recurrent cholecystitis due to occlusion of stents because we washed the gallbladder several times with 20 mL of sterile saline when EGS was performed. Stent migration at 8 months was caused by strong intestinal peristalsis, so we inserted the next stent as far as the fundus of the gallbladder because the previous stent had only reached the neck of the gallbladder. Subsequently, none of the stents have fallen out. We think that it is important to insert the stent as far as the fundus of the gallbladder and wash the gallbladder several times with sterile saline when EGS is performed to prevent stent migration and occlusion. In 1 patient (Patient no. 5), the stent was removed at 3.5 years after EGS because of a liver abscess without cholecystitis. The major cause of liver abscess may have been inadequate flow in the common bile duct because EST was not performed since that patient was on antiplatelet therapy. We recommend performing EST if possible and opening the stent side holes if that is impossible to obtain adequate flow in the common bile duct after EGS.

In 1984, Kozarek [25] introduced endoscopic transpapillary cannulation of the gallbladder. Subsequently, new catheters and guidewires have been developed to facilitate selective cannulation of the cystic duct and gallbladder [10,12,20,25]. Several other authors have attempted EGS and ETGBD in patients with acute cholecystitis [9-25]. The most recent evidenced-based report on ETGBD by Hirota et al. recommends it as an option for gallbladder drainage [2]. Clinical success was achieved in all 31 patients (100%) with acute cholecystitis by performing EGS within 3 days. Moreover, after successful emergency EGS, patients were discharged from hospital within 1 week. Thus, EGS may shorten the duration of hospitalization compared with PTGBD or ETGBD.

We consider that EGS and ETGBD are best indicated for patients with acute cholecystitis in whom transhepatic route cannot be used. Ferritis et al. [9], Toyota et al. [10], Kjaer et al. [11], and Itoi et al. [12] reported ETGBD success rates of 89% (16/18), 82% (18/22), 70.6% (24/34), and 83.7% (38/43) in smaller series. Our success rate for emergency EGS was 77.5% (31/40), which is similar to the success rate for ETGBD. We believe that EGS is slightly more difficult than ETGBD because the stent is harder to manipulate compared with an ETGBD tube (a 5Fr soft drain tube is often used for ETGBD). We encountered 3 cases in which we could not place a stent in the gallbladder despite inserting the guidewire, because of cystic duct occlusion by stones in 2 cases and severe kinking of the cystic duct in 1 case. Moreover, emergency EGS for acute cholecystitis is more difficult than elective EGS because the cystic duct often shows stricture or obstruction in acute patients. In our series, the success rate of emergency EGS was 77.5% (31/40) and this was lower than that of elective EGS (100%, 6/6).

EGS has not become very popular so far. The main reason is difficulty in negotiating the cystic duct and gallbladder with the guide wire. We used microcatheters for 4 difficult patients, and used POCS for 1 patient. The microcatheters used in 4 cases were designed for abdominal angiography and were adapted to the EGS procedure. After the microcatheter and 0.018 inch guidewire were advanced through the ERCP cannula, we could pass severe strictures and kinks of the cystic duct in all 4 cases. Thus, a microcatheter is useful for contrast studies of the cystic duct and for crossing strictures and kinks, but more highly visible and softer microcatheters should be developed. Barkay et al. [26] reported successful cannulation of the cystic duct by visualization with the SpyGlass cholangiopancreatography system (SpyGlass Direct Visualization System; Microvasive Endoscopy, Boston Scientific, Natick, MA) with EGS in a patient with cystic duct obstruction and acute cholecystitis. In 1 of our patients, a novel EGS technique using POCS was tried. POCS enabled us to endoscopically explore the cystic duct and easily insert the guidewire into the duct by using the angulation and rotation of the GIF-XP260. In our opinion, use of a microcatheter and POCS could increase the technical success rate of EGS.

Our study showed that EGS can be effective for elderly patients with acute cholecystitis who are poor surgical candidates. Generally, EGS is associated with a higher risk of aspiration pneumonia in comparison with PTGBA because EGS is performed by peroral endoscopy. EGS should be performed as rapidly as possible and improvement of EGS technology is needed in the future.

## Conclusions

We conclude that EGS can be effective for elderly patients with acute cholecystitis who are poor surgical candidates and the stent can be left for many months to several years. However, further investigations will be needed to establish the appropriate role of EGS.

## Abbreviations

CT: Computed tomography; EGS: Endoscopic gallbladder stenting; ERC: Endoscopic retrograde cholangiography; ERCP: Endoscopic retrograde cholangiopancreatography; EST: Endoscopic sphincterotomy; ETGBD: Endoscopic transpapillary naso-gallbladder drainage; POCS: Peroral cholangioscopy; PTGBD: Percutaneous transhepatic gallbladder drainage; PTGBA: Percutaneous transhepatic gallbladder aspiration; SD: Standard deviation; US: Ultrasonography.

## Competing interests

The authors declare that there are no competing interests.

## Authors’ contributions

SM, YA, and MO contributed to research design. SM, RN, TM, MO, and YA collected the data. SM drafted the manuscript. MH, RN, TM, YA, and MO contributed to revising the manuscript. All authors approved the final version.

## Pre-publication history

The pre-publication history for this paper can be accessed here:

http://www.biomedcentral.com/1471-230X/13/65/prepub

## Supplementary Material

Additional file 1 **Standard EGS procedure.** Cannulating the bile duct and cystic duct with an over-the-wire ERCP cannula. The guide wire was advanced retrogradely through the cannula into the gallbladder and a 7Fr double pig-tail stent was then advanced. Click here for file

Additional file 2 **Difficult EGS case using a microcatheter.** We accomplished EGS by using an 0.018 inch guide wire and microcatheter to pass through a severe stricture of the cystic duct. Click here for file

Additional file 3 **EGS via peroral cholangioscopy using a GIF-XP260.** We inserted the GIF-XP260 into the common duct, explored the cystic duct, and advanced the guide wire into the gallbladder. Then we exchanged GIF-XP260 for GIF-Q260, and advanced a double pigtail stent. Click here for file
